# Comprehensive Assessment of Peripheral Blood Biomarkers in Primary Pterygium: A Prospective Case–Control Study

**DOI:** 10.3390/biom16101403

**Published:** 2026-09-25

**Authors:** Dolika D. Vasović, Dejan Rašić, Dragan Spaić, Jelena Simonović, Tanja Kalezić, Jelena Vasilijević, Igor Kovačević, Miroslav Jeremić, Ivan Marjanović, Jelena Milić, Ana Tomović, Jelica Pantelić

**Affiliations:** 1 Eye Hospital, University Clinical Centre of Serbia, 11000 Belgrade, Serbia; 2 School of Medicine, University of Belgrade, 11000 Belgrade, Serbia; 3 Department of Primary Health Care and Public Health, Faculty of Medicine, University of East Sarajevo, 73300 Foca, Bosnia and Herzegovina; 4 School of Dental Medicine, University of Belgrade, 11000 Belgrade, Serbia; jelena.simonovic@stomf.bg.ac.rs

**Keywords:** pterygium, peripheral blood biomarkers, platelet indices, ceruloplasmin, oxidative stress, ocular surface disease, case–control study, systemic inflammation

## Abstract

Pterygium is a common ocular surface disorder linked to chronic ultraviolet exposure, local inflammation, and oxidative stress, yet no reliable circulating biomarker currently guides diagnosis, activity assessment, or recurrence risk. Peripheral-blood studies addressing this gap have produced inconsistent findings across hematological, inflammatory, and micronutrient domains, often using heterogeneous case definitions and isolated marker panels. In this prospective case–control study, 30 patients with newly diagnosed, clinically active primary pterygium and 17 healthy controls frequency-matched for age and sex underwent a comprehensive peripheral blood panel comprising 46 hematological, biochemical, iron-related, inflammatory, and vitamin/metabolic-bone parameters, including osteocalcin, ceruloplasmin, and immunoglobulin E. Lesions were graded using the Maheshwari and Tan classification systems, and group and severity-stratified comparisons were corrected for multiple testing using the Benjamini–Hochberg procedure. No parameter differed significantly between groups after correction. Nominal, uncorrected differences included higher total protein, albumin, and mean platelet volume in patients with pterygium, and severity-linked patterns of higher platelet count, plateletcrit, and ceruloplasmin, and higher vitamin B12, folic acid, and serum iron in less advanced lesions. None of these nominal differences survived correction, so no single marker distinguished pterygium from controls or tracked severity. Platelet indices and ceruloplasmin showed the most consistent severity-linked pattern, but this was nominal only and hypothesis-generating, warranting evaluation in larger, adequately powered cohorts.

## 1. Introduction

Pterygium is a common ocular surface disorder characterized by wing-shaped fibrovascular proliferation of the bulbar conjunctiva onto the cornea. Its global prevalence ranges from 0.3% to over 37%, depending on latitude, occupation, and environmental exposure [1,2]. Beyond its cosmetic implications, pterygium can induce astigmatism, destabilize the tear film, and, in advanced cases, invade the visual axis, resulting in permanent visual impairment [1,3]. Surgical excision with conjunctival autografting remains the standard treatment, but postoperative recurrence continues to pose a substantial clinical challenge, and no reliable circulating biomarker currently guides prognosis or recurrence risk in routine practice.

The pathogenesis of pterygium is largely attributed to chronic ultraviolet (UV) light exposure, which damages limbal stem cells and triggers local inflammation, oxidative stress, angiogenesis, and epithelial–mesenchymal transition [1]. Genetic, epigenetic, and proteomic studies have identified dysregulated expression of p53 and K-ras, matrix metalloproteinases, several microRNAs, and oxidative-stress-related proteins such as peroxiredoxin-2 within pterygium tissue [1]. Because inflammation and oxidative stress operate at both local and systemic levels, and peripheral blood sampling is far less invasive than tissue analysis, a growing body of work has asked whether these tissue-level changes are mirrored, or predicted, by alterations in routine and specialized peripheral blood parameters.

Findings for routine hematological indices have been inconsistent. One case–control study reported significantly elevated red cell distribution width (RDW), together with lower HDL-C, in patients with pterygium compared with controls [4], whereas a subsequent grade-stratified study found no association between RDW and clinical severity, despite observing reduced eosinophil counts and lower hematocrit with increasing grade [5]. A study comparing primary pterygium, recurrent pterygium, and healthy controls likewise found no significant differences in most hemogram-derived inflammatory ratios, including the neutrophil-to-lymphocyte ratio, platelet-to-lymphocyte ratio, monocyte-to-HDL ratio, and the systemic inflammation index, with only lymphocyte count differing, and only between the recurrent pterygium and control groups [6]. Serum complement adds further complexity: complement C3, although generally within the normal range, has been reported to correlate inversely with stromal opacification (T-grade) severity in primary pterygium, consistent with modest systemic complement activation in more advanced disease, whereas C4, erythrocyte sedimentation rate, and antinuclear antibody positivity showed no such association in the same cohort [7]. Serum lipid profiles illustrate a comparable lack of consensus: elevated HDL-C and LDL-C have been reported as independent risk factors in a large population-based study, with the strongest associations among adults under 50 years and those with a normal body mass index [2], and obesity, elevated total cholesterol, and an altered HDL-C/LDL-C ratio have independently predicted pterygium risk in a separate case–control study [8], findings that contrast with the lower HDL-C reported by Kurtul et al. (2019) [4]. Taken together, these discrepancies point to considerable population-specific or methodological variability across the hematological and lipid literature.

Micronutrient, trace-element, and immune-regulatory markers have been studied far less, despite their established roles in oxidative stress and chronic inflammation. One case–control study found that patients with pterygium had significantly lower serum vitamin B12, folic acid, ferritin, and vitamin D3 than controls, together with elevated free triiodothyronine and unchanged free thyroxine and thyroid-stimulating hormone [9]. This vitamin D finding runs counter to a large Korean population-based study (KNHANES, n = 19,178), in which higher blood 25-hydroxyvitamin D was associated with significantly increased odds of pterygium. This association persisted after adjustment for sunlight exposure, age, sex, smoking, diabetes, and hypertension in both men and women [10]. The basis for this discrepancy remains unclear and may involve calcium-mediated S100 protein signaling, residual confounding by UV exposure, or population-specific differences in vitamin D metabolism [10]. Other regulatory markers with plausible but largely untested relevance to pterygium include osteocalcin, a vitamin K-dependent protein implicated in inflammatory and oxidative signaling; ceruloplasmin, the principal circulating antioxidant copper-transport protein and an acute-phase reactant; and immunoglobulin E (IgE), a marker of allergic and Th2-mediated immune activity. Osteocalcin and ceruloplasmin have not, to our knowledge, previously been examined in primary pterygium, whereas IgE has been examined once, in a large Korean population-based study in which higher total serum IgE was independently associated with increased pterygium prevalence [11]; whether this reflects allergic or Th2-mediated activity specific to pterygium remains to be clarified.

Most existing studies have examined routine hematology, inflammatory ratios, complement, lipid profiles, and micronutrient status in isolation, often using heterogeneous case definitions and inconsistent adjustment for confounders such as age, BMI, and dyslipidemia. Few investigations have evaluated a comprehensive peripheral blood panel combining complete blood count with differential, iron studies, systemic inflammatory markers, and vitamin, trace-element, and metabolic-bone status within a single, prospectively enrolled case–control cohort that explicitly excludes participants with confounding systemic disease, malnutrition, or relevant supplementation.

This prospective case–control study assessed a panel of hematological, inflammatory, iron-related, and vitamin and metabolic-bone peripheral blood markers in patients with newly diagnosed, clinically active primary pterygium and in healthy controls comparable in age and sex. Given the modest size of this single-center cohort, the aim was not to establish definitive population-level associations, but to identify markers showing preliminary evidence of association with primary pterygium under strict exclusion of confounding systemic disease, and to highlight candidate markers for validation in larger, adequately powered studies.

## 2. Materials and Methods

### 2.1. Study Design and Ethical Approval

A prospective case–control study was conducted at the Eye Hospital, University Clinical Center of Serbia, Belgrade, Serbia. Approval was obtained from the Central Ethical Board of the University Clinical Center of Serbia (approval number 1787/29, dated 27 November 2025), and the study adhered to the principles of the Declaration of Helsinki. Written informed consent was obtained from all participants before enrollment.

### 2.2. Participants

Two groups were recruited from patients attending routine ophthalmological examinations: those with primary pterygium and healthy controls. Diagnosis and group allocation were determined based on clinical findings at slit-lamp biomicroscopy. Eligibility for the pterygium group required a first-time diagnosis of primary, clinically active pterygium, with no prior surgical treatment for the condition. Clinical activity was defined as a binary, examiner-based judgment at slit-lamp biomicroscopy: lesions were considered active when dilated, engorged vessels were visibly present within the pterygium. The same ophthalmologist who performed the grading made this determination for all participants. No formal hyperemia grading scale or photographic reference was used, and slit-lamp photographs were not taken. Controls were selected at recruitment to be comparable to patients in age and sex (group-level frequency matching); individual case–control pairs were not formed, and no fixed matching tolerance was predefined. Both groups were formed before laboratory results were available. The groups did not differ significantly in age or sex distribution (Table 1). Individuals were excluded from either group if they had any systemic condition known to influence peripheral blood parameters, including endocrine, hematological, immune-mediated, or rheumatological disorders, active malignancy, and malnutrition, or if they were receiving hormone, vitamin, or iron supplementation at enrollment. Patients with recurrent pterygium, pseudopterygium, or inactive or atrophic lesions were excluded, as were those with chronic ocular comorbidities such as glaucoma, uveitis, corneal or retinal disease, or a history of prior ocular surgery. A total of 30 patients with newly diagnosed primary pterygium and 17 controls without pterygium met the eligibility criteria and were included in the analysis.

### 2.3. Clinical Examination and Grading

All participants underwent comprehensive ophthalmic examination, including best-corrected visual acuity testing, slit-lamp biomicroscopy of the anterior segment and dilated fundus examination. Demographic and clinical data, such as sex, eye laterality, and lesion location, were recorded for each patient. A complete review of past and current medical history was conducted to confirm eligibility. Lesion morphology was graded according to the classification of Tan et al. [12], based on the visibility of episcleral vessels beneath the pterygium body. Lesions were categorized as T1 (atrophic, episcleral vessels clearly visible), T2 (intermediate, vessels partly visible), or T3 (fleshy, vessels fully obscured by fibrovascular tissue). No T1 (atrophic) lesions were observed in this cohort, consistent with the inclusion criterion of clinically active primary pterygium; all included lesions were therefore classified as either T2 or T3. The extent of corneal involvement was graded separately using the system of Maheshwari [3], which divides the corneal surface between the limbus and pupillary margin into two zones. Grade 1 lesions reached only the midpoint between limbus and pupil, while Grade 2 lesions extended past this midpoint toward the pupillary margin. The corresponding nasal or temporal pupillary margin was used as the reference point depending on pterygium location; in this cohort, all lesions were located on the nasal conjunctiva. All diagnoses were made, and all lesions graded, by a single experienced ophthalmologist using slit-lamp biomicroscopy during the clinical examination; in patients with pterygium, grading was performed preoperatively. Participants subsequently attended separately for blood sampling, and laboratory results became available only after grading was completed; the examiner was therefore unaware of the laboratory values. The examiner could not be masked to group status, as case definition was based on the clinical finding itself. No second examiner confirmed diagnoses or grades, and intra- and inter-observer agreement were not assessed.

### 2.4. Blood Sampling and Laboratory Analysis

Venous blood samples were collected by technicians from all participants after a 12 h overnight fast, with morning collection. Samples were processed and analyzed at the central laboratory of the University Clinical Center of Serbia, where personnel received only sample identification numbers and had no information on diagnosis, group allocation, or lesion grade. Complete blood count parameters were measured, including white cell differential (lymphocytes, monocytes, neutrophils, eosinophils, basophils), red cell indices (RBC, hemoglobin, hematocrit, MCV, MCH, MCHC, RDW), and platelet indices (platelet count, mean platelet volume, plateletcrit). Serum electrolytes and general biochemistry were assessed, including total protein, albumin, sodium, potassium, chloride, calcium, phosphorus, magnesium, bicarbonate, and serum osmolality. Iron status was evaluated by measuring serum iron, unsaturated iron-binding capacity (UIBC), total iron-binding capacity (TIBC), ferritin, and transferrin. Systemic inflammatory status was determined using interleukin-6 (IL-6) and C-reactive protein (CRP). Vitamin, trace-element, and metabolic bone status were assessed through serum vitamin D, osteocalcin, vitamin B12, folic acid, homocysteine, immunoglobulin E (IgE), and ceruloplasmin.

### 2.5. Statistical Analysis

Statistical analysis was performed using IBM SPSS Statistics, version 29.0 (IBM Corp., Armonk, NY, USA). The distribution of continuous variables was assessed separately within each comparison group using the Shapiro–Wilk test together with visual inspection of histograms and Q–Q plots. Because formal tests of normality have limited power in small samples, distributional decisions were not based on the Shapiro–Wilk test alone. Approximately normally distributed variables were presented as mean ± standard deviation and compared using Welch’s independent-samples *t*-test, which was selected because of its robustness to unequal group sizes and variances. Non-normally distributed variables were presented as median (minimum–maximum) and compared using the Mann–Whitney U test. Categorical variables were presented as n (%) and analyzed using the chi-square test or Fisher’s exact test, as appropriate. All statistical tests were two-sided. Effect sizes were expressed as Hedges’ g for comparisons performed using Welch’s *t*-test and rank-biserial correlation (rrb) for Mann–Whitney U tests, with positive values indicating higher values in the first-listed group. Ninety-five percent confidence intervals for effect sizes were estimated using bias-corrected and accelerated (BCa) bootstrap resampling with 10,000 iterations. These confidence intervals refer to the individual unadjusted effect-size estimates and were not adjusted for multiple comparisons. To account for multiple testing, the Benjamini–Hochberg false-discovery-rate procedure was applied separately to three prespecified families of 46 laboratory comparisons: the primary pterygium-versus-control analysis, the Maheshwari grade analysis, and the Tan morphology analysis. The latter two were considered exploratory severity analyses. A corrected q-value < 0.05 was considered statistically significant, whereas findings with raw *p* < 0.05 but q ≥ 0.05 were considered nominal and hypothesis-generating. As a sensitivity analysis, the Benjamini–Hochberg procedure was additionally applied simultaneously across all 138 laboratory comparisons.

## 3. Results

### 3.1. Study Population

A total of 47 participants were included in the study, comprising 30 patients with primary active pterygium and 17 controls. There were no significant differences between the groups in age (69.37 ± 8.13 vs. 71.00 ± 7.66 years; *p* = 0.496) or sex distribution (male: 56.7% vs. 41.2%; Fisher’s exact *p* = 0.371). All pterygia were located nasally. According to the Maheshwari classification, 11 lesions (36.7%) were Grade 1 and 19 (63.3%) were Grade 2. Based on Tan morphology, 18 lesions (60.0%) were classified as T2 intermediate and 12 (40.0%) as T3 fleshy (Table 1).

### 3.2. Primary Laboratory Comparison

In the unadjusted analysis, patients with pterygium had higher total protein concentrations than controls (73.23 ± 5.15 g/L vs. 69.47 ± 4.50 g/L; *p* = 0.013; Hedges’ g = 0.75) and higher albumin concentrations (45.73 ± 2.49 g/L vs. 43.76 ± 3.01 g/L; *p* = 0.030; g = 0.72). Mean platelet volume was also higher in patients with pterygium (9.75 fL (7.30–14.30) vs. 8.70 fL (7.40–10.30); *p* = 0.024; rank-biserial correlation, rrb = 0.40) (Figure 1). However, none of these differences remained statistically significant after Benjamini–Hochberg correction for 46 laboratory comparisons (all q = 0.460). No additional laboratory parameter showed a nominal between-group difference at *p* < 0.05 (Table 2A–C).

### 3.3. Exploratory Analysis by Maheshwari Grade

Patients with Maheshwari Grade 1 and Grade 2 pterygium were comparable in age (69.45 ± 8.81 vs. 69.32 ± 7.95 years; *p* = 0.966) and sex distribution (male: 54.5% vs. 57.9%; Fisher’s exact *p* = 1.000). In the unadjusted analysis, patients with Grade 1 lesions had higher vitamin B12 concentrations [306.0 ng/L (206.0–551.0) vs. 227.0 ng/L (103.0–360.0); exact *p* = 0.006], higher folic acid concentrations [7.90 ug/L (4.90–14.63) vs. 5.10 ug/L (3.60–21.39); exact *p* = 0.026], and higher serum iron levels (17.89 ± 3.24 umol/L vs. 15.16 ± 3.72 umol/L; *p* = 0.046). None of these differences remained statistically significant after the Benjamini–Hochberg correction (q = 0.276, 0.598, and 0.705, respectively). The complete results of all 46 laboratory comparisons are provided in Supplementary Table S1.

### 3.4. Exploratory Analysis by Tan Morphology

Patients with T2 intermediate and T3 fleshy pterygium did not differ significantly in age (67.56 ± 7.49 vs. 72.08 ± 8.61 years; *p* = 0.152) or sex distribution (male: 66.7% vs. 41.7%; Fisher’s exact *p* = 0.264). In the unadjusted analysis, patients with T3 lesions had higher platelet counts (256.4 ± 57.5 10^9^/L vs. 200.6 ± 41.8 10^9^/L; *p* = 0.009), plateletcrit values (0.251 ± 0.067% vs. 0.192 ± 0.042%; *p* = 0.015), ceruloplasmin concentrations [0.290 g/L (0.250–0.430) vs. 0.245 g/L (0.190–0.330); exact *p* = 0.002], and CRP-to-albumin ratios [0.074 (0.013–0.170) vs. 0.027 (0.013–0.068); exact *p* = 0.043]. Conversely, serum iron (17.42 ± 3.19 umol/L vs. 14.28 ± 3.84 umol/L; *p* = 0.030) and transferrin saturation (32.71 ± 6.89% vs. 25.86 ± 8.81%; *p* = 0.035) were higher in patients with T2 lesions. None of these differences remained statistically significant following Benjamini–Hochberg correction (all q ≥ 0.092). The complete results of all 46 laboratory comparisons are provided in Supplementary Table S2. Nominally significant findings from both exploratory severity analyses are summarized in Table 3.

Across the primary analysis and both exploratory severity analyses, no laboratory parameter met the prespecified FDR-adjusted significance threshold of q < 0.05. Accordingly, findings observed at the unadjusted level should be interpreted as exploratory and hypothesis-generating.

The Maheshwari and Tan classification systems were not significantly associated in this sample. Among Grade 1 lesions, 9/11 (81.8%) were T2 and 2/11 (18.2%) were T3, whereas among Grade 2 lesions, 9/19 (47.4%) were T2 and 10/19 (52.6%) were T3 (Fisher’s exact *p* = 0.121; Cramér’s V = 0.34).

Across the primary analysis and both exploratory severity analyses, no laboratory parameter met the prespecified FDR-adjusted significance threshold of q < 0.05. A sensitivity analysis in which the Benjamini–Hochberg procedure was applied simultaneously across all 138 laboratory comparisons yielded the same conclusion; no comparison remained statistically significant, with the smallest global FDR-adjusted q-value being 0.276. Accordingly, findings observed at the unadjusted level should be interpreted as exploratory and hypothesis-generating.

## 4. Discussion

This prospective case–control study examined a broad panel of hematological, biochemical, iron-related, inflammatory, and vitamin/metabolic-bone parameters in patients with newly diagnosed, clinically active primary pterygium and in age- and sex-comparable controls. After correction for multiple comparisons, none of the circulating parameters distinguished pterygium patients from controls. At the unadjusted level, patients with pterygium showed nominally higher total protein, albumin, and mean platelet volume (MPV) than controls. Exploratory analyses stratified by severity identified further associations: vitamin B12, folic acid, and serum iron varied with Maheshwari grade, while platelet indices, ceruloplasmin, serum iron, transferrin saturation, and the CRP-to-albumin ratio varied with Tan morphology. None of these differences withstood Benjamini–Hochberg correction and should therefore be regarded as exploratory and hypothesis-generating rather than confirmed findings.

This pattern of largely negative systemic findings contrasts with the local, tissue- and tear-based literature, in which pterygium has repeatedly been associated with measurable abnormalities linked to disease activity and severity. He and Wu (2022) summarized numerous genetic, epigenetic, and proteomic alterations within pterygium tissue, including dysregulated p53 and K-ras expression, matrix metalloproteinases, and oxidative-stress-related proteins such as peroxiredoxin-2 [1]. At the genomic level, Detorakis et al. (1998) reported loss of heterozygosity in more than half of excised pterygia, most frequently at chromosomal regions 9p and 17q, with correlations to clinical variables [13]. Vascular remodeling within the lesion has also been well-characterized: Martín-López et al. (2019) reported an approximately threefold-higher blood-to-lymphatic vessel ratio in pterygium than in normal conjunctiva, with vascular endothelial growth factor (VEGF)-A expression exceeding VEGF-C, consistent with angiogenesis rather than lymphangiogenesis as the dominant local vascular process [14]. Han et al. (2025) similarly demonstrated, using optical coherence tomography angiography and functional slit-lamp biomicroscopy, that ocular surface vessel density increases progressively from control to mild to severe pterygium, with vessel dilation predominating in milder lesions and vessel branching in more severe lesions [15]. However, hemodynamic flow parameters did not differ significantly between groups. Reda et al. (2018) showed that clinical redness correlates with histopathological vascularity, epithelial hyperplasia, and lymphocytic stromal infiltration [16], while Lan et al. (2024) found that tear lymphotoxin-alpha, a cytokine of the tumor necrosis factor superfamily, is significantly lower in clinically active than inactive pterygium and negatively correlates with lesion size [17]. Recent studies have extended this picture. Niu et al. (2026) combined anterior segment OCT with the systemic immune-inflammation index (SII) in 90 patients with pterygium. They found that the SII correlated with lesion area and excised tissue weight, and that the imaging data allowed for pterygium volume to be estimated [18]. Genetic association studies in a Taiwanese population linked the MMP-11 rs738792 C allele [19] and the MMP-3 rs3025058 5A allele [20] to increased pterygium risk, whereas the MMP-13 variants rs2252070 and rs478927 showed no association [21]. At the tissue level, granzyme B-positive cells, many of which co-expressed the mast cell marker tryptase, were more numerous in pterygium than in control conjunctiva [22]; M1 macrophage markers were upregulated and linked to VEGF-C expression, whereas expression of the M2 marker CD163 correlated inversely with clinical severity [23]; expression of PAX6 and DSG1 was reduced in the corneal epithelium of patients with pterygium [24]; and SPARC was reported to promote angiogenesis and cell migration through a VEGFR2-ERK1/2-SPHK1 axis [25]. Collectively, these findings indicate that pterygium is associated with distinct, severity- and activity-related molecular and structural changes at the level of the lesion and adjacent ocular surface. Our results suggest that these local changes are not necessarily reflected in generalized alterations in peripheral blood, at least in a cohort of this size, and this distinction between local and systemic findings provides a useful framework for interpreting the results discussed below.

Among routine hematological indices, RDW did not differ significantly between groups and was numerically lower in patients with pterygium (Hedges’ g = −0.48). This is notable because Kurtul et al. (2019) reported significantly higher RDW in pterygium patients than in controls and proposed RDW as an inflammatory and oxidative-stress marker relevant to pterygium pathogenesis [4]. In contrast, Shukla et al. (2026) subsequently found no significant association between RDW and pterygium severity in a larger, grade-stratified series, despite observing lower eosinophil counts and a negative correlation between hematocrit and severity grade [5]. Our study likewise found no significant differences in eosinophil count or hematocrit when comparing pterygium with controls or across severity strata. The directionally opposite RDW findings across these studies illustrate how a single hematological index can appear as a significant risk marker in one cohort and show no association, or even the opposite trend, in another, underscoring the sensitivity of such indices to population characteristics, comparator-group definitions, and sample size.

Mean platelet volume was nominally higher in patients with pterygium than in controls in the unadjusted analysis, although this difference did not survive correction for multiple testing. Neither Kurtul et al. (2019) [4] nor Alimoğlu et al. (2026) [6], who compared primary pterygium, recurrent pterygium, and healthy controls across a wide panel of hemogram-derived inflammatory indices, found a significant difference in platelet-related parameters between groups. In our severity-stratified analysis, however, platelet count and plateletcrit were nominally higher in fleshy (Tan T3) than in intermediate (Tan T2) lesions. This finding parallels the vascular observations of Han et al. (2025) [15], who showed that ocular surface vessel density and morphology change progressively with pterygium severity even though systemic hemodynamic flow parameters remained unchanged; in both cases, a severity-linked structural or cellular signal was present while a broader physiological parameter was not. Because MPV is an established marker of platelet reactivity, and larger, more reactive platelets have been associated with angiogenic and inflammatory activity in other vascular conditions, this severity-linked platelet signal is consistent with the possibility that platelet activation reflects the degree of local vascular and fibrovascular activity within a pterygium lesion rather than the presence or absence of the disease itself. This distinction between disease presence and disease morphology may help explain why studies comparing pterygium broadly against controls have produced inconsistent platelet findings, whereas morphology-stratified comparisons may prove more informative; this possibility warrants testing in larger, adequately powered, severity-stratified cohorts.

Hemogram-derived systemic inflammatory ratios, including the neutrophil-to-lymphocyte ratio (NLR), platelet-to-lymphocyte ratio (PLR), monocyte-to-lymphocyte ratio (MLR), and systemic immune-inflammation index (SII), together with interleukin-6 and C-reactive protein, did not differ significantly between patients with pterygium and controls in our cohort. This is broadly consistent with the results of Alimoğlu et al. (2026) [6], who reported no significant differences in NLR, PLR, eosinophil-to-lymphocyte ratio, monocyte-to-eosinophil ratio, MLR, monocyte-to-HDL ratio, or SII across primary pterygium, recurrent pterygium, and healthy controls; only lymphocyte count differed significantly, and only between the recurrent pterygium and control groups. In contrast, Niu et al. (2026) recently reported significantly higher SII, NLR, and MLR values in patients with pterygium than in controls and found the SII to be associated with pterygium severity and with lesion area and volume assessed using anterior-segment imaging and AS-OCT [18]. In our cohort, NLR, PLR, MLR, and SII values did not differ significantly between patients with pterygium and controls, and the SII was not significantly different between T2 and T3 lesions. Differences in sample size and phenotyping may partly explain these discrepant findings, as Niu et al. studied 90 patients and incorporated quantitative assessment of pterygium area and volume, whereas our severity analyses were based on clinical Maheshwari and Tan classifications. These contrasting findings underscore the need for larger studies integrating systemic inflammatory indices with quantitative lesion imaging. In our exploratory analysis, the CRP-to-albumin ratio was nominally higher in fleshy (T3) than in intermediate (T2) lesions. Although this specific marker has not previously been examined in pterygium, the direction of this association is conceptually consistent with the results of Yun et al. (2025) [7], who found that although erythrocyte sedimentation rate and most autoimmune markers were not associated with pterygium severity, serum complement C3 correlated inversely with stromal opacification (T grade), consistent with modest systemic complement activation in more advanced disease. It is also consistent with the histopathological observation of Reda et al. (2018) that inflammatory cell density was not significantly correlated with any clinical parameter in pterygium tissue, even though vascularity and epithelial hyperplasia were [16]. Together, these findings suggest that vascular and structural features, rather than inflammatory cell density itself, may be the more reliable correlates of pterygium severity, both locally and systemically. At the level of immune cell populations, a Mendelian randomization analysis suggested that a higher proportion of CD39+ CD8+ T cells within the T cell compartment is causally associated with pterygium risk (odds ratio = 1.19) [26], which raises the possibility that immune contributions are better resolved at the level of specific cell subsets than by global blood indices such as those measured here. Complement components were not included in our panel; incorporating C3 and C4 alongside the CRP-to-albumin ratio in future severity-stratified studies could help clarify whether a shared, low-grade systemic inflammatory or complement-mediated process accompanies more advanced, fleshier lesion morphology. Proteomic and functional evidence supports the idea that C5a/C5aR signaling may contribute to pterygium pathogenesis by promoting epithelial–mesenchymal transition-related changes in conjunctival epithelial cells [27], complementing the C3-severity association reported by Yun et al. [7].

Vitamin D, vitamin B12, folic acid, and ferritin did not differ significantly between patients with pterygium and controls in our overall comparison. This contrasts with the results of Sumer et al. (2025) [9], who reported significantly lower levels of all four markers in pterygium patients than in matched controls. It does not replicate the positive association between higher blood 25-hydroxyvitamin D and pterygium reported by Jee et al. (2016) in the large Korean National Health and Nutrition Examination Survey (KNHANES) cohort [10]. This association persisted after adjustment for sunlight exposure and other confounders. In our exploratory Maheshwari-grade analysis, however, patients with less advanced (Grade 1) pterygium had higher vitamin B12, folic acid, and serum iron than those with more advanced (Grade 2) lesions, a pattern compatible with progressive micronutrient depletion accompanying more active tissue proliferation, and broadly consistent with the overall depletion reported by Sumer et al. (2025) [9], even though that study compared pterygium with controls rather than across severity grades. Given the opposite-direction vitamin D findings reported by Sumer et al. (2025) [9] and Jee et al. (2016) [10], and the absence of any vitamin D signal in our own data at either the group or severity level, the relationship between micronutrient status and pterygium is unlikely to be uniform across populations; it probably depends on comparator-group definition, disease activity, sample size, and regional baseline micronutrient status, and cannot yet be considered established. Trace elements involved in antioxidant defense have also been examined: in the Korean National Health and Nutrition Examination Survey population, higher urinary arsenic was associated with pterygium (odds ratio 1.84 for the highest versus the lowest tertile), the second tertile of blood manganese showed lower odds than the lowest (odds ratio 0.53), and serum zinc was not associated with pterygium [28]. Because our panel excluded trace elements, such as ceruloplasmin, a copper-transport protein, future panels combining arsenic, manganese, zinc, and ceruloplasmin could test the oxidative-stress hypothesis more directly.

To our knowledge, this is the first study to examine osteocalcin and ceruloplasmin in primary pterygium. Immunoglobulin E (IgE) was included to test for potential systemic Th2/allergic activation in our cohort, an association previously reported by Yoo et al. (2018) in a large Korean population-based study [11], in which higher total serum IgE was independently associated with a greater prevalence of pterygium. Ceruloplasmin, the principal circulating copper-transport antioxidant and an acute-phase reactant, was nominally higher in fleshy (T3) than in intermediate (T2) lesions in our cohort, which may reflect mobilization of a copper-dependent antioxidant defence against the greater oxidative burden of more vascularized, actively proliferating tissue, consistent with the central role of oxidative stress in pterygium pathogenesis described by He and Wu (2022) [1] and with the angiogenesis-dominant vascular profile reported in advanced lesions by Martín-López et al. (2019) [14]. Copper metabolism has also been implicated at the transcriptomic level: an integrative bioinformatic analysis of pterygium tissue identified cuproptosis-related genes, which belong to a copper-dependent cell death pathway, whose dysregulation was linked to immune responses, and downregulation of selected candidate biomarkers was confirmed in pterygium tissue compared with normal conjunctiva, although the authors regard these findings as hypothesis-generating [29]. Because these data relate to tissue rather than serum, it is unclear whether circulating ceruloplasmin reflects these copper-related processes. In our own cohort, however, IgE showed no association with pterygium at either the group or severity level, a discrepancy with Yoo et al. (2018) [11] that may reflect our considerably smaller sample size, differences in age distribution, or differences in IgE thresholds and confounder adjustment between the two studies, rather than a genuine absence of association. Osteocalcin likewise showed no signal at either the group or severity level, providing no support for a role of the vitamin K-dependent bone-inflammatory axis in this population. However, our modest sample size limits the ability to exclude smaller effects for either marker. All three markers warrant further investigation in larger cohorts, particularly ceruloplasmin, given its association with fleshy lesion morphology here.

The most unexpected finding was a nominal elevation in total protein and albumin in patients with pterygium relative to controls, an association that, to our knowledge, has not been previously reported. Because acute-phase inflammatory responses typically lower rather than raise albumin, this finding does not fit a simple acute-phase model of pterygium-associated systemic inflammation, and more plausibly reflects a nonspecific group difference in hydration status, dietary protein intake, or another unmeasured confounder. As it did not survive correction for multiple testing, we regard it as hypothesis-generating only, and recommend that future studies account for hydration and nutritional status when comparing protein-related parameters between pterygium patients and controls.

Across the primary comparison and both exploratory severity analyses, none of the 46 laboratory parameters met the pre-specified false-discovery-rate threshold of q < 0.05. This partly reflects the combination of a modest sample size (n = 47, including only 30 pterygium cases) with a wide multi-domain panel, a substantial multiple-testing burden in comparison with the epidemiological studies that first reported significant vitamin D associations, which enrolled tens of thousands of participants [10]. More broadly, the peripheral-blood pterygium literature is characterized by real heterogeneity: RDW has been reported as elevated [4] or unassociated with severity [5], vitamin D has been reported as both positively [10] and inversely [9] associated with pterygium, and systemic inflammatory ratios have been reported as elevated in some studies but unchanged in others [6,7]. This mirrors the heterogeneity already documented at the genetic, epigenetic, and proteomic levels by He and Wu (2022) [1] and at the genomic level by Detorakis et al. (1998) [13], where numerous candidate tissue biomarkers and loci of genetic instability have been identified, but none have achieved validated clinical utility. Even within the matrix metalloproteinase family, variants of MMP-11 and MMP-3 but not MMP-13 have been associated with pterygium risk [19,20,21]. Differences in case definition (primary versus recurrent, clinically active versus atrophic), severity grading systems, the extent of exclusion of confounding systemic disease, and population characteristics such as age, BMI, ethnicity, and cumulative UV exposure likely all contribute to this inconsistency.

This study has several limitations. The overall sample size was modest, and recruitment was single-center, limiting statistical power and generalizability to other populations. The exploratory severity subgroups were particularly small (Maheshwari Grade 1 vs. Grade 2: n = 11 vs. 19; Tan T2 vs. T3: n = 18 vs. 12), resulting in limited precision and relatively wide confidence intervals; therefore, nominal subgroup findings should be interpreted as hypothesis-generating. The small sample sizes also limit the ability of formal normality tests to reliably detect departures from normality, which is why Shapiro–Wilk testing was supplemented by visual assessment of histograms and Q–Q plots and non-parametric testing was used where appropriate. In addition, several laboratory variables were biologically or mathematically related, including erythrocyte indices, platelet measures, and iron-status parameters. Consequently, the 46 laboratory comparisons did not represent completely independent biological hypotheses. Although the Benjamini–Hochberg procedure was applied to control the false discovery rate within each prespecified analysis family, this dependency structure should be considered when interpreting the adjusted results. Importantly, an additional sensitivity analysis applying the Benjamini–Hochberg procedure simultaneously across all 138 laboratory comparisons yielded the same overall conclusion, with no comparison remaining statistically significant. The case–control comparison was based on a single time-point biomarker assessment and therefore does not permit causal inference. Our cohort was older, on average, than many previously published pterygium series, which may limit comparability with studies reporting age-dependent associations that are strongest in younger participants. A single examiner performed diagnosis and grading and could not be blinded to group status; without independent confirmation or reliability testing, observer bias in the severity classifications cannot be ruled out. However, the examiner was unaware of laboratory results, and laboratory staff were unaware of clinical status, which limits this risk. In addition, clinical activity was defined by a subjective slit-lamp assessment of visible dilated, engorged vessels, without a formal grading scale, photographic reference, or independent confirmation, so some misclassification of activity cannot be excluded. Controls were matched only approximately on age and sex, and not on other potentially relevant factors such as BMI, dyslipidemia, or cumulative UV exposure, which the sample size did not allow us to adjust for. We did not obtain tissue-level or tear-fluid correlates, such as the vascular, genomic, or cytokine markers described by Martín-López [14], Detorakis [13], and Lan [17], nor longitudinal recurrence data, and the sample size precluded meaningful multivariable adjustment for potential confounders. Strengths include prospective design, preoperative grading of all lesions by a single experienced examiner using two validated classification systems [3,12], mutual blinding between clinical assessment and laboratory analysis, and the stringent exclusion of participants with systemic disease, malnutrition, or relevant supplementation, and prespecified correction for multiple comparisons rather than selective reporting of nominally significant findings. Larger, multicentre cohorts stratified by age, BMI, and pterygium clinical activity or severity will be needed before the nominal associations identified here, particularly the severity-linked patterns involving platelet indices, ceruloplasmin, and micronutrient status, can be considered candidate biomarkers for pterygium activity, prognosis, or recurrence risk. Incorporating complement components [7], tear-based inflammatory markers such as lymphotoxin-alpha [17], and quantitative ocular surface vascular imaging [15] alongside a systemic panel could help clarify whether the well-documented local abnormalities of pterygium are ever reflected in peripheral blood. Longitudinal follow-up through and beyond surgical excision would further allow these markers to be evaluated as predictors of postoperative recurrence, which remains the most pressing unmet clinical need in pterygium management. Fibrosis is thought to contribute to postoperative recurrence, and researchers are exploring fibrotic pathways as therapeutic targets: the Rho kinase inhibitor Y27632 reduced viability, migration, and the expression of extracellular matrix and myofibroblast markers in cultured primary pterygium cells [30]. Whether circulating markers relate to such fibrotic or postoperative phenotypes remains to be examined and could be addressed in longitudinal designs.

## 5. Conclusions

In this prospective case–control study of a broad peripheral blood panel, no single hematological, biochemical, inflammatory, or micronutrient marker distinguished patients with newly diagnosed, clinically active primary pterygium from healthy controls after correction for multiple comparisons. Nominal, uncorrected signals, higher total protein, albumin, and mean platelet volume overall, together with severity-linked patterns of higher platelet count, plateletcrit, and ceruloplasmin and lower serum iron and transferrin saturation in fleshy versus intermediate lesions, and higher vitamin B12, folic acid, and serum iron in less advanced lesions, are hypothesis-generating only and should be interpreted cautiously given the modest sample size and the absence of correction-surviving associations.

Considered alongside the marked inconsistency of previously reported hematological, inflammatory, and micronutrient associations across the pterygium literature, these findings suggest that no peripheral blood marker currently has sufficient evidence to serve as a standalone biomarker of pterygium presence, activity, or severity in routine practice. Platelet indices and ceruloplasmin, which showed the most consistent severity-linked signal in our exploratory analyses, together with complement components highlighted in prior work, represent the most promising candidates for evaluation in larger, adequately powered, multicentre cohorts with harmonized case definitions, severity grading, and confounder adjustment.

## Figures and Tables

**Figure 1:**
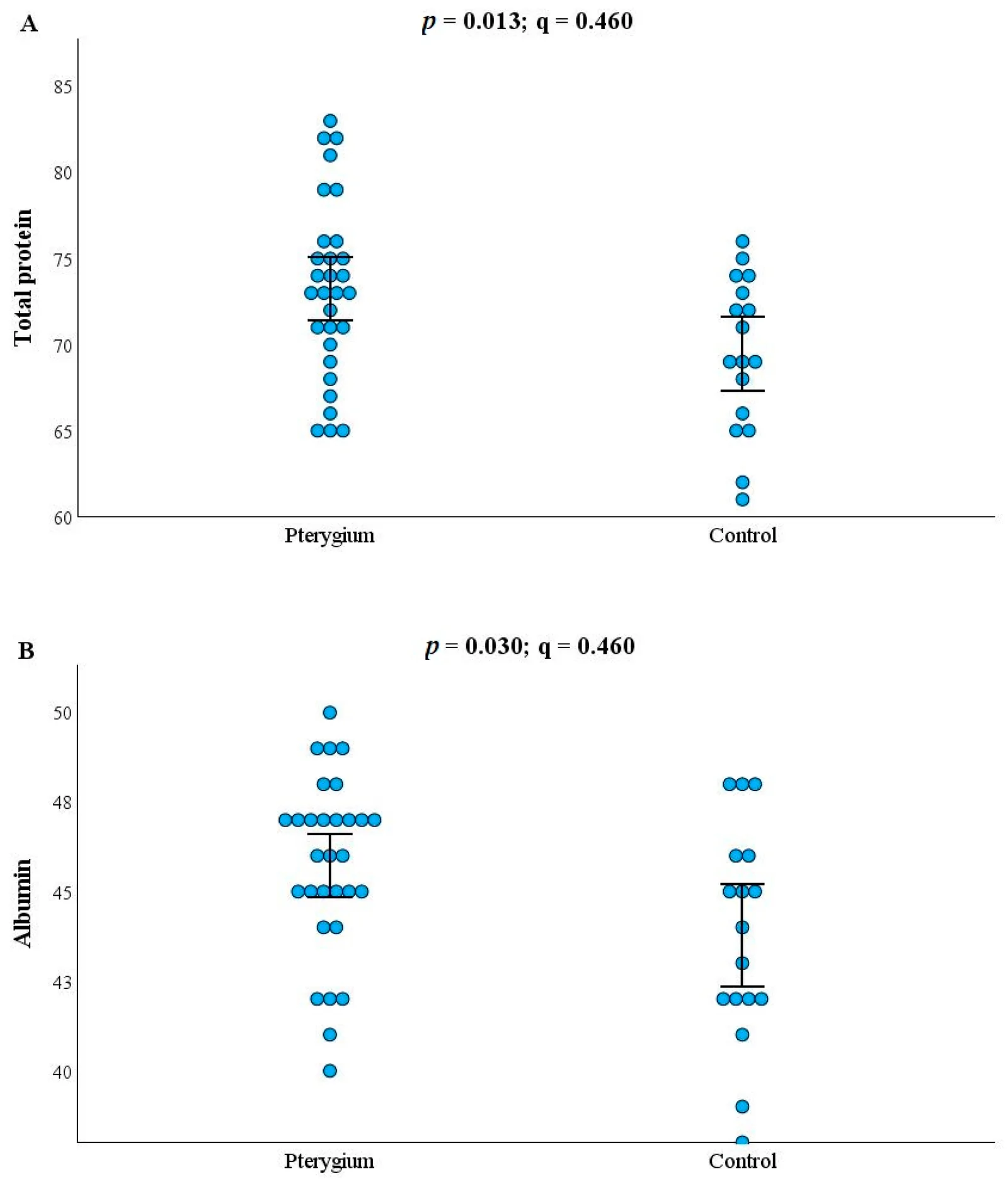

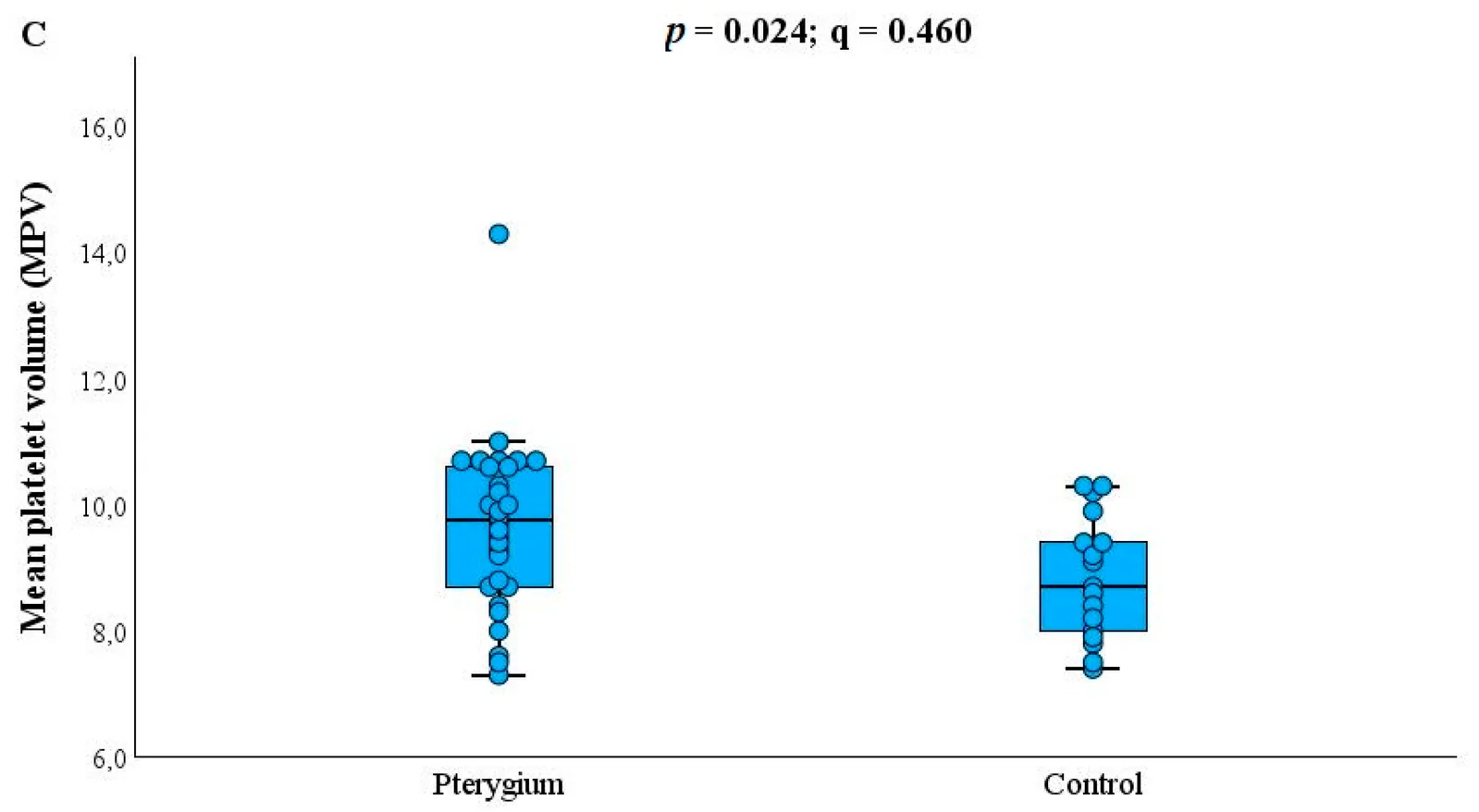
Distribution of total protein (**A**), albumin (**B**), and mean platelet volume (**C**) in patients with primary pterygium and controls. Individual observations are shown for all parameters. Total protein and albumin are summarized by the mean and 95% confidence interval, whereas MPV is displayed using a box-and-whisker plot. *p*-values are unadjusted two-sided values; q-values were obtained using the Benjamini–Hochberg procedure.

**Table 1:** Demographic and clinical characteristics.

Characteristic	Primary Pterygium (n = 30)	Controls (n = 17)	*p*-Value
Age, years	69.37 ± 8.13	71.00 ± 7.66	0.496
Male	17 (56.7%)	7 (41.2%)	0.371
Female	13 (43.3%)	10 (58.8%)	
Nasal location	30 (100.0%)	-	-
Maheshwari Grade 1	11 (36.7%)	-	-
Maheshwari Grade 2	19 (63.3%)	-	-
Tan T2 intermediate	18 (60.0%)	-	-
Tan T3 fleshy	12 (40.0%)	-	-

Data are mean ± SD or n (%). Age was compared using Welch’s *t*-test and sex using Fisher’s exact test.

**Table 2:** (**A**) Complete blood count and derived inflammatory indices: primary pterygium versus controls. (**B**) Biochemical, electrolyte, and iron-status parameters: primary pterygium versus controls. (**C**) Inflammatory, vitamin, and metabolic markers: primary pterygium versus controls.

Parameter	Primary Pterygium (n = 30)	Controls (n = 17)	Effect Size (95% CI)	Raw *p*	FDR q
(A)					
White blood cell count (WBC) (10^9^/L)	7.36 ± 2.01	6.44 ± 1.47	g = 0.49 (−0.04–1.04)	0.078	0.512
Lymphocytes (10^9^/L)	2.04 ± 0.60	1.90 ± 0.65	g = 0.21 (−0.43–0.82)	0.491	0.956
Monocytes (10^9^/L)	0.59 (0.24–1.21)	0.51 (0.31–1.04)	r_rb_ = 0.26 (−0.11–0.59)	0.135	0.537
Neutrophils (10^9^/L)	4.50 ± 1.49	3.74 ± 1.16	g = 0.53 (−0.07–1.07)	0.062	0.512
Eosinophils (10^9^/L)	0.16 (0.02–0.47)	0.15 (0.02–0.82)	r_rb_ = −0.02 (−0.39–0.35)	0.929	0.974
Basophils (10^9^/L)	0.04 (0.00–0.10)	0.04 (0.02–0.11)	r_rb_ = −0.13 (−0.46–0.21)	0.446	0.956
Red blood cell count (RBC) (10^12^/L)	4.61 ± 0.44	4.61 ± 0.47	g = 0.02 (−0.61–0.61)	0.956	0.974
Hemoglobin (g/L)	137.5 (123.0–175.0)	135.0 (118.0–158.0)	r_rb_ = 0.08 (−0.28–0.42)	0.634	0.974
Hematocrit (L/L)	0.406 (0.358–0.518)	0.406 (0.364–0.470)	r_rb_ = 0.01 (−0.35–0.35)	0.974	0.974
Mean corpuscular volume (MCV) (fL)	88.66 ± 4.09	88.75 ± 4.74	g = −0.02 (−0.62–0.62)	0.952	0.974
Mean corpuscular hemoglobin (MCH) (pg)	29.99 ± 1.57	29.62 ± 1.75	g = 0.22 (−0.40–0.82)	0.475	0.956
Mean corpuscular hemoglobin concentration (MCHC) (g/L)	338.33 ± 7.64	333.82 ± 7.37	g = 0.59 (−0.03–1.17)	0.055	0.512
Red cell distribution width (RDW) (%)	13.28 ± 0.93	13.70 ± 0.69	g = −0.48 (−1.04–0.10)	0.089	0.512
Platelet count (PLT) (10^9^/L)	222.9 ± 55.3	251.1 ± 58.7	g = −0.49 (−1.11–0.13)	0.116	0.534
Mean platelet volume (MPV) (fL)	9.75 (7.30–14.30)	8.70 (7.40–10.30)	r_rb_ = 0.40 (0.06–0.66)	0.024	0.460
Plateletcrit (PCT) (%)	0.215 ± 0.060	0.222 ± 0.055	g = −0.10 (−0.70–0.48)	0.724	0.974
Neutrophil-to-lymphocyte ratio (NLR)	2.32 ± 0.82	2.18 ± 1.04	g = 0.15 (−0.54–0.78)	0.646	0.974
Platelet-to-lymphocyte ratio (PLR)	109.37 (65.28–214.14)	122.34 (75.50–251.16)	r_rb_ = −0.33 (−0.62–0.04)	0.068	0.512
Monocyte-to-lymphocyte ratio (MLR)	0.32 (0.11–0.51)	0.28 (0.18–0.61)	r_rb_ = 0.13 (−0.26–0.47)	0.479	0.956
Systemic immune-inflammation index (SII)	485.68 (195.83–1067.83)	472.19 (215.17–1512.14)	r_rb_ = 0.02 (−0.34–0.36)	0.938	0.974
(B)					
Total protein (g/L)	73.23 ± 5.15	69.47 ± 4.50	g = 0.75 (0.16–1.30)	0.013	0.460
Albumin (g/L)	45.73 ± 2.49	43.76 ± 3.01	g = 0.72 (0.08–1.40)	0.030	0.460
Sodium (mmol/L)	139.5 (136.0–148.0)	140.0 (129.0–144.0)	r_rb_ = −0.05 (−0.41–0.32)	0.771	0.974
Potassium (mmol/L)	4.49 ± 0.47	4.43 ± 0.41	g = 0.13 (−0.46–0.70)	0.664	0.974
Chloride (mmol/L)	101.90 ± 3.12	101.94 ± 3.77	g = −0.01 (−0.66–0.63)	0.970	0.974
Calcium (mmol/L)	2.42 (2.20–2.61)	2.41 (1.26–2.62)	r_rb_ = 0.26 (−0.11–0.57)	0.140	0.537
Phosphorus (mmol/L)	1.05 ± 0.20	1.05 ± 0.11	g = 0.02 (−0.49–0.55)	0.944	0.974
Magnesium (mmol/L)	0.87 ± 0.05	0.84 ± 0.07	g = 0.47 (−0.15–1.08)	0.157	0.556
Bicarbonate (mmol/L)	25.0 (21.0–33.0)	26.0 (23.0–29.0)	r_rb_ = −0.10 (−0.41–0.23)	0.569	0.974
Serum osmolality (mOsm/kg)	275.5 (208.0–298.0)	255.0 (212.0–293.0)	r_rb_ = 0.29 (−0.07–0.58)	0.106	0.534
Serum iron (ug/L)	16.16 ± 3.74	14.68 ± 4.27	g = 0.37 (−0.26–1.02)	0.242	0.742
Unsaturated iron-binding capacity (UIBC) (umol/L)	38.88 ± 8.53	40.18 ± 7.04	g = −0.16 (−0.75–0.43)	0.577	0.974
Total iron-binding capacity (TIBC) (umol/L)	55.04 ± 6.61	54.85 ± 7.01	g = 0.03 (−0.62–0.62)	0.929	0.974
Ferritin (ug/L)	120.50 (16.90–400.00)	89.30 (37.10–813.00)	r_rb_ = 0.12 (−0.26–0.46)	0.492	0.956
Transferrin (g/L)	2.52 ± 0.28	2.50 ± 0.32	g = 0.08 (−0.58–0.69)	0.810	0.974
Transferrin saturation (%)	29.97 ± 8.30	26.93 ± 7.86	g = 0.37 (−0.27–0.98)	0.221	0.726
(C)					
Interleukin-6 (IL-6) (pg/mL)	3.95 (1.50–32.10)	3.70 (1.50–22.00)	r_rb_ = 0.06 (−0.28–0.40)	0.731	0.974
C-reactive protein (CRP) (mg/L)	1.50 (0.60–7.50)	1.60 (0.60–3.70)	r_rb_ = 0.07 (−0.27–0.41)	0.681	0.974
CRP-to-albumin ratio	0.033 (0.013–0.170)	0.036 (0.012–0.097)	r_rb_ = 0.03 (−0.30–0.37)	0.877	0.974
25-hydroxyvitamin D (nmol/L)	58.50 (18.00–150.00)	61.00 (42.00–148.00)	r_rb_ = −0.12 (−0.45–0.24)	0.499	0.956
Osteocalcin (ug/L)	21.00 (11.00–56.00)	22.00 (13.00–72.00)	r_rb_ = −0.08 (−0.41–0.26)	0.642	0.974
Vitamin B12 (ng/L)	255.0 (103.0–551.0)	284.0 (118.0–758.0)	r_rb_ = −0.19 (−0.53–0.20)	0.293	0.812
Folic acid (ug/L)	6.32 (3.60–21.39)	6.20 (2.60–18.29)	r_rb_ = 0.02 (−0.36–0.39)	0.903	0.974
Homocysteine (umol/L)	16.00 (9.00–32.00)	15.00 (10.00–24.00)	r_rb_ = 0.05 (−0.29–0.38)	0.764	0.974
Immunoglobulin E (IgE) (IU/mL)	25.50 (10.00–154.00)	25.00 (4.50–85.00)	r_rb_ = 0.13 (−0.25–0.48)	0.472	0.956
Ceruloplasmin (g/L)	0.26 (0.19–0.43)	0.25 (0.19–0.31)	r_rb_ = 0.18 (−0.16–0.49)	0.300	0.812

Values are mean ± SD for Welch *t*-tests and median (minimum–maximum) for Mann–Whitney U tests. Mann–Whitney *p*-values are exact when available. Effect sizes are Hedges g or rank-biserial correlation (r_rb_); positive values indicate higher values in the first-listed group. Ninety-five percent confidence intervals for effect sizes were estimated using bias-corrected and accelerated (BCa) bootstrap resampling with 10,000 iterations. Confidence intervals refer to the unadjusted individual effect-size estimates and were not adjusted for multiple comparisons. q-values were calculated with the Benjamini–Hochberg procedure across 46 comparisons.

**Table 3:** Exploratory laboratory findings according to pterygium severity.

Classification	Parameter	Grade 1	Grade 2	Effect Size (95% CI)	Raw *p*	FDR q
Maheshwari	Vitamin B12 (ng/L)	306.0 (206.0–551.0)	227.0 (103.0–360.0)	r_rb_ = 0.60 (0.17–0.85)	0.006	0.276
	Folic acid (ug/L)	7.90 (4.90–14.63)	5.10 (3.60–21.39)	r_rb_ = 0.49 (0.06–0.79)	0.026	0.598
	Serum iron (umol/L)	17.89 ± 3.24	15.16 ± 3.72	g = 0.75 (0.08–1.40)	0.046	0.705
		T2 intermediate	T3 fleshy			
Tan morphology	Ceruloplasmin (g/L)	0.245 (0.190–0.330)	0.290 (0.250–0.430)	r_rb_ = −0.66 (−0.88 to−0.28)	0.002	0.092
	Platelet count (PLT) (10^9/L)	200.6 ± 41.8	256.4 ± 57.5	g = −1.12 (−1.96 to −0.17)	0.009	0.207
	Plateletcrit (PCT) (%)	0.192 ± 0.042	0.251 ± 0.067	g = −1.08 (−2.09 to −0.02)	0.015	0.230
	Serum iron (umol/L)	17.42 ± 3.19	14.28 ± 3.84	g = 0.88 (0.16–1.61)	0.030	0.322
	Transferrin saturation (%)	32.71 ± 6.89	25.86 ± 8.81	g = 0.86 (0.07–1.63)	0.035	0.322
	CRP-to-albumin ratio	0.027 (0.013–0.068)	0.074 (0.013–0.170)	r_rb_ = −0.44 (−0.80–0.05)	0.043	0.330

Values are mean ± SD for Welch *t*-tests and median (minimum–maximum) for Mann–Whitney U tests. Mann–Whitney *p*-values are exact when available. Effect sizes are Hedges g or rank-biserial correlation (r_rb_); positive values indicate higher values in the first-listed group. Ninety-five percent confidence intervals for effect sizes were estimated using bias-corrected and accelerated (BCa) bootstrap resampling with 10,000 iterations. Confidence intervals refer to the unadjusted individual effect-size estimates and were not adjusted for multiple comparisons. q-values were calculated with the Benjamini–Hochberg procedure across 46 comparisons.

## Data Availability

Data are contained within the article.

## Supplementary Materials

The following supporting information can be downloaded at https://www.mdpi.com/article/10.3390/biom16101403/s1, Table S1: Complete laboratory comparison according to Maheshwari grade; Table S2: Complete laboratory comparison according to Tan morphology.

## References

[1] He S., Wu Z. (2022). Biomarkers in the occurrence and development of pterygium. Ophthalmic Res..

[2] Zang S., Chen Y., Guo H., Zhang M., Zhang G., Zhang L., Liu Q., Zeng J., Lo C.H., Cui Y. (2022). High HDL-C and high LDL-C are risk factors of pterygium in a population-based cross-sectional study in Southern China: The Dongguan Eye Study. BMJ Open.

[3] Maheshwari S. (2007). Pterygium-induced corneal refractive changes. Indian J. Ophthalmol..

[4] Kurtul B.E., Kabatas E.U., Ozates S. (2019). The correlation of routine hematological indices with pterygium. Ther. Adv. Ophthalmol..

[5] Shukla R., Garg P., Shrinkhal, Shukla M., Shukla A., Nahar R., Singh S.P., Mishra A.K., Banerjee N. (2026). Exploring the link between hematological parameters and ocular surface disease: Insights from pterygium. Int. J. Appl. Basic Med. Res..

[6] Alimoğlu S.A., Çakır B., Özata Gündoğdu K. (2026). The evaluation of hemogram-related systemic inflammatory markers in pterygium recurrence. BMC Ophthalmol..

[7] Yun Y.I., Heo J.S., Lee S.H., Kim K.W. (2025). Association of serum complement C3 level with disease severity in primary pterygium. Front. Med..

[8] Sun Y., Liang W., Zeng Y., Luo S., Huang C., Liu Y. (2023). The role of serum lipids and BMI in China patients with primary pterygium. Precis. Med. Sci..

[9] Sumer F., Kizilkaya B., Yildiz E., Satilmaz M.F., Suveys A. (2025). Evaluation of the role of thyroid hormones, vitamin B12, vitamin D3, folic acid and ferritin serum levels in pterygium development. Sci. Rep..

[10] Jee D., Kim E.C., Cho E., Arroyo J.G. (2016). Positive association between blood 25-hydroxyvitamin D levels and pterygium after control for sunlight exposure. PLoS ONE.

[11] Yoo T.K., Kim S.W., Seo K.Y. (2018). Association between serum immunoglobulin E and pterygium: A population-based study from South Korea. Curr. Eye Res..

[12] Tan D.T.H., Chee S.P., Dear K.B.G., Lim A.S.M. (1997). Effect of pterygium morphology on pterygium recurrence in a controlled trial comparing conjunctival autografting with bare sclera excision. Arch. Ophthalmol..

[13] Detorakis E.T., Sourvinos G., Tsamparlakis J., Spandidos D.A. (1998). Evaluation of loss of heterozygosity and microsatellite instability in human pterygium: Clinical correlations. Br. J. Ophthalmol..

[14] Martín-López J., Pérez-Rico C., García-Honduvilla N., Buján J., Pascual G. (2019). Elevated blood/lymphatic vessel ratio in pterygium and its relationship with vascular endothelial growth factor (VEGF) distribution. Histol. Histopathol..

[15] Han J., Gong Q., Zhu X., Ji R., Li M., Wang H., Qu J., Hu L. (2025). Vascular characteristics and haemodynamics of severe and mild pterygium: A quantitative analysis. Ann. Med..

[16] Reda A.M., Shaaban Y.M.M., Saad El-Din S.A. (2018). Histopathological parameters in pterygia and significant clinical correlations. J. Ophthalmic Vis. Res..

[17] Lan G., Fang X., Zhong Y., Luo S., Xiao X., Xie Z., Luo L., Zhang Y., Li H., Lin Y. (2024). Evaluation of lymphotoxin-alpha in pterygium and diagnostic value in active and inactive pterygium states. Sci. Rep..

[18] Niu R., Jin X., Yan Z., Zhao Z., Fan L., Du Z., Wang L., Liu J., An J., Wang X. (2026). A new approach to assessing pterygium progression and volume using SII and anterior segment OCT. Front. Med..

[19] Hsia N.Y., Chen H.C., Tsai H.W., Hsia T.C., Hu P.S., Wang Y.C., Shih H.Y., Chang W.S., Cheng D.C., Bau D.T. (2024). Impacts of matrix metalloproteinase-11 genotypes on pterygium risk. Anticancer Res..

[20] Chen H.C., Hsia N.Y., Tsai C.L., Hsia T.C., Hu P.S., Wang Y.C., Shih H.Y., Chang W.S., Lin J.C., Bau D.T. (2026). Unravelling the contribution of matrix metalloproteinase-3 genotype-phenotype to pterygium risk. Cancer Genom. Proteom..

[21] Chen H.C., Hsia N.Y., Hu P.S., Hsia T.C., Wang Y.C., Shih H.Y., Chang W.S., Bau D.T., Tsai C.W. (2025). Exploring the genetic role of matrix metalloproteinase-13 variants in pterygium risk. In Vivo.

[22] Choi Y., Samad I., Chakravarthy H., Matsubara J., Granville D.J., Yeung S.N. (2024). Granzyme B expression in conjunctiva of patients with pterygium. Int. J. Mol. Sci..

[23] Lee S.J., Koh A., Lee S.H., Kim K.W. (2024). Distinct activation of M1 and M2 macrophages in the primary pterygium lymphangiogenesis. Exp. Eye Res..

[24] Stachon T., Fries F.N., Li Z., Daas L., Nagy Z.Z., Seitz B., Szentmáry N. (2025). Decreased PAX6 and DSG1 protein expression in corneal epithelium of patients with epithelial basal membrane dystrophy, Salzmann nodular degeneration, and pterygium. J. Clin. Med..

[25] Xu Y., Yu D., Wang L., Lou J., Zhuang X., Chen Y., Lin W., Zhang X. (2026). SPARC promotes angiogenesis and cell migration via VEGFR2-ERK1/2-SPHK1 axis in pterygium. Exp. Eye Res..

[26] Yang J., Liang B.Y., Li J.J., Fang C.Y., Li Y., Liu H. (2025). Immunological determinants of pterygium pathogenesis: Mendelian randomization analysis of CD39+ CD8+ T cell proportions within the T cell compartment. Medicine.

[27] Han J., Gong Q., Wang H., Xie B., Ye L., Shen L., Hu L. (2026). Complement C5a promotes epithelial-mesenchymal transition in pterygium via C5aR activation. Investig. Ophthalmol. Vis. Sci..

[28] Han K., Kim K.W., Choi Y.H. (2025). Associations of urine arsenic, blood manganese, and serum zinc with pterygium in Korean adults. J. Trace Elem. Med. Biol..

[29] Yang J., Liang B.Y., Fang C.Y., Li J.J., Zhang P., Xiang P., Zhang L.W., Liu H. (2026). Exploring dysregulation of cuproptosis-related genes molecular clusters and candidate biomarkers in pterygium. Sci. Rep..

[30] Dai J., Rayana N.P., Peng M., Sugali C.K., Harvey D.H., Dhamodaran K., Yu E., Dalloul J.M., Liu S., Mao W. (2024). Inhibition of pterygium cell fibrosis by the Rho kinase inhibitor. Sci. Rep..

