# Supplementary material for: Comprehensive Assessment of Peripheral Blood Biomarkers in Primary Pterygium: A Prospective Case–Control Study
Source: Biomolecules. 2026 Sep 25;16(10):1403. doi: 10.3390/biom16101403 (PMC13702147; doi:10.3390/biom16101403)
Supplement: Supplementary file 1 [file biomolecules-16-01403-s001.zip › biomolecules-4554088-supplementary.pdf]

Supplementary Materials

Table S1. Complete laboratory comparison according to Maheshwari grade

Parameter	Grade 1 (n=11)	Grade 2 (n=19)	Effect size (95% CI)	Raw p	FDR q
Complete blood count and derived inflammatory indices					
White blood cell count (WBC) (10^9/L)	7.48±2.12	7.29±2.01	g = 0.09 (-0.65-0.87)	0.810	1.000
Lymphocytes (10^9/L)	2.03±0.61	2.04±0.61	g = -0.03 (-0.75-0.75)	0.944	1.000
Monocytes (10^9/L)	0.63±0.27	0.61±0.18	g = 0.08 (-0.73-0.97)	0.844	1.000
Neutrophils (10^9/L)	4.58±1.46	4.45±1.55	g = 0.09 (-0.63-0.85)	0.813	1.000
Eosinophils (10^9/L)	0.19±0.13	0.14±0.09	g = 0.42 (-0.35-1.23)	0.313	1.000
Basophils (10^9/L)	0.04±0.02	0.04±0.02	g = -0.32 (-1.03-0.38)	0.364	1.000
Red blood cell count (RBC) (10^{12}/L)	4.67±0.57	4.58±0.36	g = 0.20 (-0.66-1.06)	0.642	1.000
Hemoglobin (g/L)	140.0±15.0	136.9±8.9	g = 0.26 (-0.55-1.10)	0.549	1.000
Hematocrit (L/L)	0.413±0.043	0.405±0.027	g = 0.22 (-0.58-1.08)	0.600	1.000
Mean corpuscular volume (MCV) (fL)	88.71±3.64	88.64±4.43	g = 0.02 (-0.70-0.74)	0.962	1.000
Mean corpuscular hemoglobin (MCH) (pg)	30.30 (26.50-32.40)	29.30 (28.10-33.20)	r_{rb} = 0.11 (-0.35-0.51)	0.641	1.000
Mean corpuscular hemoglobin concentration (MCHC) (g/L)	338.73±8.27	338.11±7.47	g = 0.08 (-0.64-0.89)	0.839	1.000
Red cell distribution width (RDW) (%)	12.88±0.86	13.52±0.91	g = -0.69 (-1.39 to-0.005)	0.071	0.816

Platelet count (PLT) (10^9/L)	213.6±66.1	228.3±49.1	g = -0.26 (-1.04-0.65)	0.532	1.000
Mean platelet volume (MPV) (fL)	9.70 (7.60-14.30)	9.80 (7.30-11.00)	r_{rb} = 0.005 (-0.44-0.44)	1.000	1.000
Plateletcrit (PCT) (%)	0.207±0.061	0.220±0.061	g = -0.21 (-0.97-0.55)	0.567	1.000

Parameter	Grade 1 (n=11)	Grade 2 (n=19)	Effect size (95% CI)	Raw p	FDR q
Neutrophil-to-lymphocyte ratio (NLR)	2.37±0.75	2.29±0.87	g = 0.09 (-0.61-0.85)	0.794	1.000
Platelet-to-lymphocyte ratio (PLR)	108.23±25.86	118.81±36.19	g = -0.31 (-0.90-0.41)	0.361	1.000
Monocyte-to-lymphocyte ratio (MLR)	0.32±0.10	0.31±0.09	g = 0.04 (-0.72-0.84)	0.919	1.000
Systemic immune-inflammation index (SII)	482.67 (195.83-760.48)	488.69 ([illegible]45.03-1067.83)	r_{rb} = 0.01 (-0.44-0.45)	0.966	1.000
Biochemical, electrolyte, and iron-status parameters					
Total protein (g/L)	74.91±3.18	72.26±5.87	g = 0.51 (-0.16-1.24)	0.120	0.920
Albumin (g/L)	46.18±2.23	45.47±2.65	g = 0.27 (-0.48-0.96)	0.442	1.000
Sodium (mmol/L)	139.0 (136.0-148.0)	140.0 (136.0-146.0)	r_{rb} = -0.02 (-0.47-0.45)	0.933	1.000
Potassium (mmol/L)	4.55±0.44	4.45±0.49	g = 0.19 (-0.52-0.94)	0.599	1.000
Chloride (mmol/L)	102.00±3.46	101.84±3.00	g = 0.05 (-0.72-0.86)	0.901	1.000
Calcium (mmol/L)	2.44±0.07	2.42±0.11	g = 0.17 (-0.46-0.84)	0.599	1.000
Phosphorus (mmol/L)	1.01±0.26	1.07±0.17	g = -0.29 (-1.13-0.51)	0.494	1.000

Magnesium (mmol/L)	0.88±0.03	0.86±0.06	g = 0.42 (-0.24-1.04)	0.206	1.000
Bicarbonate (mmol/L)	24.0 (22.0-27.0)	26.0 (21.0-33.0)	r_{rb} = -0.18 (-0.56-0.25)	0.445	1.000
Serum osmolality (mOsm/kg)	275.0 (246.0-296.0)	277.0 (208.0-298.0)	r_{rb} = -0.005 (-0.41-0.42)	1.000	1.000
Serum iron (ug/L)	17.89±3.24	15.16±3.72	g = 0.75 (0.08-1.4)	0.046	0.705
Unsaturated iron-binding capacity (UIBC) (umol/L)	36.65±7.95	40.17±8.80	g = -0.40 (-1.07-0.39)	0.272	1.000
Total iron-binding capacity (TIBC) (umol/L)	54.54±6.62	55.33±6.78	g = -0.12 (-0.80-0.70)	0.756	1.000
Ferritin (ug/L)	138.00 (26.70-400.00)	111.00 (16.90-316.00)	r_{rb} = 0.17 (-0.29-0.55)	0.471	1.000
Transferrin (g/L)	2.54±0.28	2.51±0.28	g = 0.12 (-0.64-0.90)	0.748	1.000
Parameter	Grade 1 (n=11)	Grade 2 (n=19)	Effect size (95% CI)	Raw p	FDR q
Transferrin saturation (%)	33.33±7.78	28.02±8.15	g = 0.64 (-0.04-1.31)	0.091	0.837
Inflammatory, vitamin, and metabolic markers					
Interleukin-6 (IL-6) (pg/mL)	3.80 (1.50-32.10)	4.10 (1.50-23.60)	r_{rb} = 0.01 (-0.42-0.43)	0.966	1.000
C-reactive protein (CRP) (mg/L)	2.10 (0.70-3.30)	0.90 (0.60-7.50)	r_{rb} = 0.19 (-0.23-0.56)	0.395	1.000
CRP-to-albumin ratio	0.045 (0.015-0.080)	0.021 (0.013-0.170)	r_{rb} = 0.16 (-0.27-0.55)	0.471	1.000
25-hydroxyvitamin D (nmol/L)	60.00 (38.00-78.00)	57.00 (18.00-150.00)	r_{rb} = 0.005 (-0.41-0.42)	1.000	1.000
Osteocalcin (ug/L)	20.00 (11.00-47.00)	22.00 (11.00-56.00)	r_{rb} = -0.16 (-0.57-0.30)	0.471	1.000
Vitamin B12 (ng/L)	306.0 (206.0-551.0)	227.0 (103.0-360.0)	r_{rb} = 0.60 (0.17-0.85)	0.006	0.276

Folic acid (ug/L)	7.90 (4.90-14.63)	5.10 (3.60-21.39)	r_{rb} = 0.49 (0.06-0.79)	0.026	0.598
Homocysteine (umol/L)	15.00 (10.00-22.00)	16.00 (9.00-32.00)	r_{rb} = -0.15 (-0.55-0.27)	0.497	1.000
Immunoglobulin E (IgE) (IU/mL)	39.00 (16.00-143.00)	23.00 (10.00-154.00)	r_{rb} = 0.23 (-0.23-0.60)	0.307	1.000
Ceruloplasmin (g/L)	0.250 (0.220-0.330)	0.260 (0.190-0.430)	r_{rb} = -0.14 (-0.53-0.28)	0.525	1.000

Values are mean ± SD for Welch t-tests and median (minimum–maximum) for Mann–Whitney U tests. Mann–Whitney p-values are exact when available. Effect sizes are Hedges g or rank-biserial correlation (r_{rb}); positive values indicate higher values in the first-listed group. Ninety-five 5 percent confidence intervals for effect sizes were estimated using bias-corrected and accelerated (BCa) bootstrap resampling with 10,000 iterations.
Confidence intervals refer to the unadjusted individual effect-size estimates and were not adjusted for multiple comparisons. q-values were calculated with the Benjamini–Hochberg procedure across 46 comparisons.

Table S2. Complete laboratory comparison according to Tan morphology

Parameter	T2 intermediate (n=18)	T3 fleshy (n=12)	Effect size	Raw p	FDR q
Complete blood count and derived inflammatory indices					
White blood cell count (WBC) (10^9/L)	7.01±1.40	7.89±2.68	g = -0.43 (-1.31-0.44)	0.308	0.714
Lymphocytes (10^9/L)	1.95±0.50	2.17±0.74	g = -0.35 (-1.14-0.49)	0.388	0.741
Monocytes (10^9/L)	0.59±0.16	0.66±0.28	g = -0.36 (-1.13-0.49)	0.391	0.741
Parameter	T2 intermediate (n=18)	T3 fleshy (n=12)	Effect size	Raw p	FDR q
Neutrophils (10^9/L)	4.25±1.12	4.87±1.92	g = -0.41 (-1.24-0.45)	0.326	0.714
Eosinophils (10^9/L)	0.18 (0.06-0.47)	0.11 (0.02-0.39)	r_{rb} = 0.29 (-0.18-0.66)	0.185	0.567
Basophils (10^9/L)	0.04±0.02	0.05±0.03	g = -0.27 (-1.14-0.58)	0.523	0.776
Red blood cell count (RBC) (10^{12}/L)	4.56±0.44	4.69±0.45	g = -0.28 (-1.02-0.44)	0.451	0.741
Hemoglobin (g/L)	137.5 (123.0-157.0)	137.5 (125.0-175.0)	r_{rb} = 0.06 (-0.38-0.46)	0.787	0.953

Hematocrit (L/L)	0.406 (0.358-0.446)	0.408 (0.365-0.518)	r_{rb} = 0.05 (-0.39-0.45)	0.851	0.986
Mean corpuscular volume (MCV) (fL)	89.30±4.41	87.71±3.54	g = 0.38 (-0.35-1.07)	0.284	0.714
Mean corpuscular hemoglobin (MCH) (pg)	30.10 (26.50-33.20)	28.90 (28.10-31.10)	r_{rb} = 0.36 (-0.10-0.69)	0.104	0.399
Mean corpuscular hemoglobin concentration (MCHC) (g/L)	339.50±8.31	336.58±6.43	g = 0.37 (-0.33-1.05)	0.289	0.714
Red cell distribution width (RDW) (%)	13.18±0.95	13.43±0.93	g = -0.26 (-0.99-0.50)	0.481	0.756
Platelet count (PLT) (10^9/L)	200.6±41.8	256.4±57.5	g = -1.12 (-1.96 to-0.17)	0.009	0.207
Mean platelet volume (MPV) (fL)	9.45 (7.50-14.30)	9.90 (7.30-11.00)	r_{rb} = -0.18 (-0.59-0.27)	0.415	0.741
Plateletcrit (PCT) (%)	0.192±0.042	0.251±0.067	g = -1.08 (-2.09 to-0.02)	0.015	0.230
Neutrophil-to-lymphocyte ratio (NLR)	2.31±0.80	2.33±0.87	g = -0.02 (-0.81-0.74)	0.949	0.993
Platelet-to-lymphocyte ratio (PLR)	106.76±25.56	127.18±39.24	g = -0.63 (-1.38-0.12)	0.130	0.460
Monocyte-to-lymphocyte ratio (MLR)	0.31±0.09	0.31±0.10	g = 0.004 (-0.75-0.77)	0.991	1.000
Systemic immune-inflammation index (SII)	452.92±155.36	599.97±266.97	g = -0.69 (-1.46-0.09)	0.104	0.399
Biochemical, electrolyte, and iron-status parameters					
Total protein	72.61±5.01 (g/L)	74.17±5.44	g = -0.29 (-1.04-0.45)	0.437	0.741
Albumin	47.00	44.50	r_{rb} = 0.37	0.095	0.399
Parameter	T2 intermediate (n=18)	T3 fleshy (n=12)	Effect size	Raw p	FDR q
(g/L)	(40.00-49.00)	(41.00-50.00)	(-0.17-0.76)		

Sodium (mmol/L)	139.0 (136.0-148.0)	140.0 (136.0-146.0)	r_{rb} = -0.17 (-0.57-0.26)	0.439	0.741
Potassium (mmol/L)	4.30 (4.00-5.30)	4.75 (3.30-5.20)	r_{rb} = -0.31 (-0.69-0.17)	0.172	0.565
Chloride (mmol/L)	101.83±2.46	102.00±4.05	g = -0.05 (-0.92-0.76)	0.900	0.986
Calcium (mmol/L)	2.40±0.07	2.47±0.12	g = -0.77 (-1.71-0.28)	0.077	0.394
Phosphorus (mmol/L)	1.05±0.23	1.06±0.17	g = -0.06 (-0.73-0.66)	0.871	0.986
Magnesium (mmol/L)	0.87 (0.74-0.95)	0.87 (0.79-0.98)	r_{rb} = -0.08 (-0.52-0.38)	0.723	0.899
Bicarbonate (mmol/L)	24.0 (21.0-29.0)	26.0 (21.0-33.0)	r_{rb} = -0.10 (-0.57-0.40)	0.662	0.870
Serum osmolality (mOsm/kg)	272.5 (208.0-296.0)	277.0 (211.0-298.0)	r_{rb} = -0.14 (-0.56-0.31)	0.545	0.783
Serum iron (ug/L)	17.42±3.19	14.28±3.84	g = 0.88 (0.16-1.61)	0.030	0.322
Unsaturated iron-binding capacity (UIBC) (umol/L)	36.35±6.49	42.67±10.04	g = -0.76 (-1.57-0.04)	0.071	0.394
Total iron-binding capacity (TIBC) (umol/L)	53.77±5.61	56.95±7.75	g = -0.47 (-1.27-0.30)	0.236	0.679
Ferritin (ug/L)	114.00 (45.60-263.00)	131.00 (16.90-400.00)	r_{rb} = -0.02 (-0.50-0.45)	0.950	0.993
Transferrin (g/L)	2.49±0.26	2.56±0.31	g = -0.26 (-1.06-0.50)	0.493	0.756
Transferrin saturation (%)	32.71±6.89	25.86±8.81	g = 0.86 (0.07-1.63)	0.035	0.322
Inflammatory, vitamin, and metabolic markers					
Interleukin-6 (IL-6) (pg/mL)	3.75 (1.50-32.10)	3.95 (1.50-23.60)	r_{rb} = -0.13 (-0.54-0.32)	0.573	0.799
C-reactive protein (CRP) (mg/L)	1.25 (0.60-3.30)	3.20 (0.60-7.50)	r_{rb} = -0.42 (-0.76-0.06)	0.053	0.348

CRP-to-albumin ratio	0.027 (0.013-0.068)	0.074 (0.013-0.170)	r_{rb} = -0.44 (-0.80-0.05)	0.043	0.330
25-hydroxyvitamin D (nmol/L)	56.50 (18.00-83.00)	64.50 (28.00-150.00)	r_{rb} = -0.22 (-0.64-0.28)	0.325	0.714

Parameter	T2 intermediate (n=18)	T3 fleshy (n=12)	Effect size	Raw p	FDR q
Osteocalcin (ug/L)	22.50 (12.00-56.00)	18.50 (11.00-46.00)	r_{rb} = 0.12 (-0.33-0.53)	0.602	0.814
Vitamin B12 (ng/L)	244.0 (103.0-551.0)	273.5 (155.0-389.0)	r_{rb} = -0.20 (-0.59-0.25)	0.368	0.741
Folic acid (ug/L)	6.46 (3.99-14.63)	5.68 (3.60-21.39)	r_{rb} = 0.09 (-0.38-0.51)	0.692	0.884
Homocysteine (umol/L)	16.00 (10.00-22.00)	15.00 (9.00-32.00)	r_{rb} = 0.00 (-0.48-0.47)	1.000	1.000
Immunoglobulin E (IgE) (IU/mL)	25.50 (10.00-102.00)	27.50 (10.00-154.00)	r_{rb} = -0.04 (-0.49-0.42)	0.884	0.986
Ceruloplasmin (g/L)	0.245 (0.190-0.330)	0.290 (0.250-0.430)	r_{rb} = -0.66 (-0.88 to-0.28)	0.002	0.092

Values are mean ± SD for Welch t-tests and median (minimum–maximum) for Mann–Whitney U tests. Mann–Whitney p-values are exact when available. Effect sizes are Hedges g or rank-biserial correlation (r_{rb}); positive values indicate higher values in the first-listed group. Ninety-five percent confidence intervals for effect sizes were estimated using bias-corrected and accelerated (BCa) bootstrap resampling with 10,000 iterations. Confidence intervals refer to the unadjusted individual effect-size estimates and were not adjusted for multiple comparisons. q-values were calculated with the Benjamini–Hochberg procedure across 46 comparisons.
